# Two-Weekly High-Dose-Rate Brachytherapy Boost After External Beam Radiotherapy for Localized Prostate Cancer: Long-Term Outcome and Toxicity Analysis

**DOI:** 10.3389/fonc.2021.764536

**Published:** 2021-11-26

**Authors:** Jörg Tamihardja, Paul Lutyj, Johannes Kraft, Dominik Lisowski, Stefan Weick, Michael Flentje, Bülent Polat

**Affiliations:** Department of Radiation Oncology, University of Würzburg, Würzburg, Germany

**Keywords:** prostate cancer, high-dose-rate (HDR) brachytherapy, radiotherapy, long-term outcome, toxicity, external beam radiotherapy (EBRT), biochemical relapse free survival

## Abstract

**Purpose:**

Evaluation of clinical outcome of two-weekly high-dose-rate brachytherapy boost after external beam radiotherapy (EBRT) for localized prostate cancer.

**Methods:**

338 patients with localized prostate cancer receiving definitive EBRT followed by a two-weekly high-dose-rate brachytherapy boost (HDR-BT boost) in the period of 2002 to 2019 were analyzed. EBRT, delivered in 46 Gy (D_Mean_) in conventional fractionation, was followed by two fractions HDR-BT boost with 9 Gy (D_90%_) two and four weeks after EBRT. Androgen deprivation therapy (ADT) was added in 176 (52.1%) patients. Genitourinary (GU)/gastrointestinal (GI) toxicity was evaluated utilizing the Common Toxicity Criteria for Adverse Events (version 5.0) and biochemical failure was defined according to the Phoenix definition.

**Results:**

Median follow-up was 101.8 months. 15 (4.4%)/115 (34.0%)/208 (61.5%) patients had low-/intermediate-/high-risk cancer according to the D`Amico risk classification. Estimated 5-year and 10-year biochemical relapse-free survival (bRFS) was 84.7% and 75.9% for all patients. The estimated 5-year bRFS was 93.3%, 93.4% and 79.5% for low-, intermediate- and high-risk disease, respectively. The estimated 10-year freedom from distant metastasis (FFM) and overall survival (OS) rates were 86.5% and 70.0%. Cumulative 5-year late GU toxicity and late GI toxicity grade ≥ 2 was observed in 19.3% and 5.0% of the patients, respectively. Cumulative 5-year late grade 3 GU/GI toxicity occurred in 3.6%/0.3%.

**Conclusions:**

Two-weekly HDR-BT boost after EBRT for localized prostate cancer showed an excellent toxicity profile with low GU/GI toxicity rates and effective long-term biochemical control.

## Introduction

Prostate cancer represents the most common cancer type among adult men (1). Curative radiotherapy in localized disease is well established. Due to a low α/β - ratio of prostate cancer and subsequent high sensitivity to dose fractionation, hypofractionated and dose-escalated therapy regimes show an improved therapeutic ratio in the treatment of prostate cancer (2–5). However, keeping the limits of normal tissue tolerance for organs at risk remains difficult in dose-escalated external beam radiation therapy (EBRT). In contrast, high-dose-rate brachytherapy (HDR-BT) is able to deliver high single doses while respecting the dose constraints of the surrounding organs at risk. HDR-BT is also not quite as affected by the movement of organs at risk caused by organ filling compared to EBRT and offers excellent dose conformity. Nevertheless, there is concern that periprostatic disease, especially in high-risk cancer, is not treated sufficiently by HDR-BT alone. To obtain the advantages of both therapies, EBRT is often combined with a HDR-BT boost and randomized data has shown the superiority of the combination therapy over EBRT alone (6, 7).

**Figure 1:**
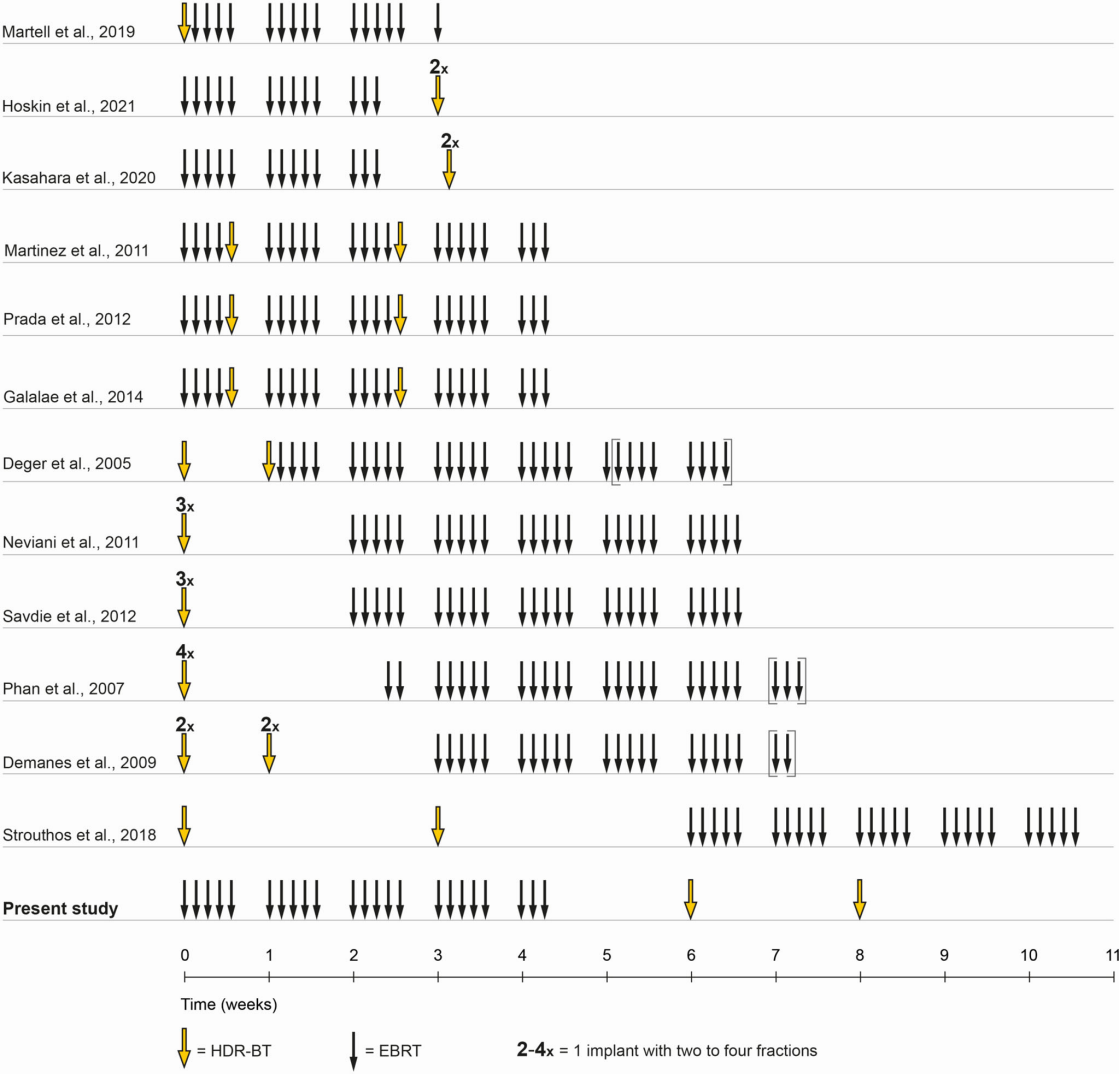
Treatment schedule comparison. Shown is a comparison of timelines of combined EBRT and HDR-BT boost in the literature. In the present study, a treatment schedule with EBRT and two implants, two fractions HDR-BT boost was chosen. EBRT was administered in 4.5 weeks in 23 fractions of each 2 Gy. The first HDR-BT boost fraction with 9 Gy was applied one to two weeks after the end of EBRT with the second fraction following two weeks after.

Up to date, no standard treatment regime of combined EBRT and HDR-BT boost exists and the GEC/ESTRO guidelines state a wide range of possible regimes mostly based on published retrospective trials (8–20). While randomized trial data on this subject remains scarce, there is limited data on two-weekly HDR-BT boost after EBRT (**Figure 1**). This current publication reports long-term biochemical relapse-free survival and presents results of genitourinary and gastrointestinal toxicity in patients with localized prostate cancer treated with EBRT in combination with two-weekly high-dose-rate brachytherapy.

## Materials and Methods

### Patient Characteristics

This retrospective single-center analysis is based on 338 consecutive male patients treated between 2002 and 2019 with combined two-weekly high-dose-rate brachytherapy boost after external beam radiotherapy (EBRT) for localized prostate cancer. All patients had pathologically confirmed prostate cancer and were stratified into risk groups according to D’Amico et al. (21). During the implementation of the treatment protocol, a small number of low-risk patients were included. Later on, low-risk patients were excluded from dose-escalation by combined EBRT and HDR brachytherapy. Additive androgen deprivation therapy was recommended for patients with intermediate-risk (6 months) and high-risk disease (24–36 months) and prescribed at the discretion of the treating urologist. Staging examinations before radiotherapy included abdominal computed tomography, digital rectal examination, transrectal ultrasound (TRUS), prostate-specific antigen (PSA) serum testing, and bone scintigraphy. Magnetic resonance imaging (MRI) was not performed regularly as MRI assessment only became an internal standard during the study period. Biochemical failure was defined according to the Phoenix definition as nadir plus a ≥ 2 ng/ml increase in the prostate-specific antigen (PSA). Assessment of physician-recorded toxicity during radiotherapy was performed at baseline, at the end of the treatment, 6 weeks after treatment, and in 6 months intervals thereafter. After two years, follow-up was changed to longer periods with annual examinations. Gastrointestinal (GI) and genitourinary (GU) toxicity were scored using Common Terminology Criteria for Adverse Events (CTCAE) v5.0. Acute toxicity was defined as occurring within 3 months after radiotherapy. Late toxicity assessment included the 6 monthly and all later follow-ups.

### External Beam Radiation Therapy

EBRT was delivered with 3D-conformal radiation therapy (3D-CRT), intensity-modulated radiation therapy (IMRT), or volumetric-modulated arc therapy (VMAT) in 23 fractions with 2 Gy per fraction, resulting in a prescribed planning target volume (PTV) dose of 46 Gy (D_Mean_). A clinical target volume (CTV) was generated consisting of the prostate and the seminal vesicles. The PTV was created by a 10 mm margin around the CTV in all but the dorsal direction, where a 7 mm margin was used. Pinnacle^3^ (Philips Radiation Oncology Systems, Fitchburg, WI, USA) was used for treatment planning. Pelvic lymph node irradiation was performed depending on the individual decision and risk stratification.

### HDR Brachytherapy Boost

**Figure 2:**
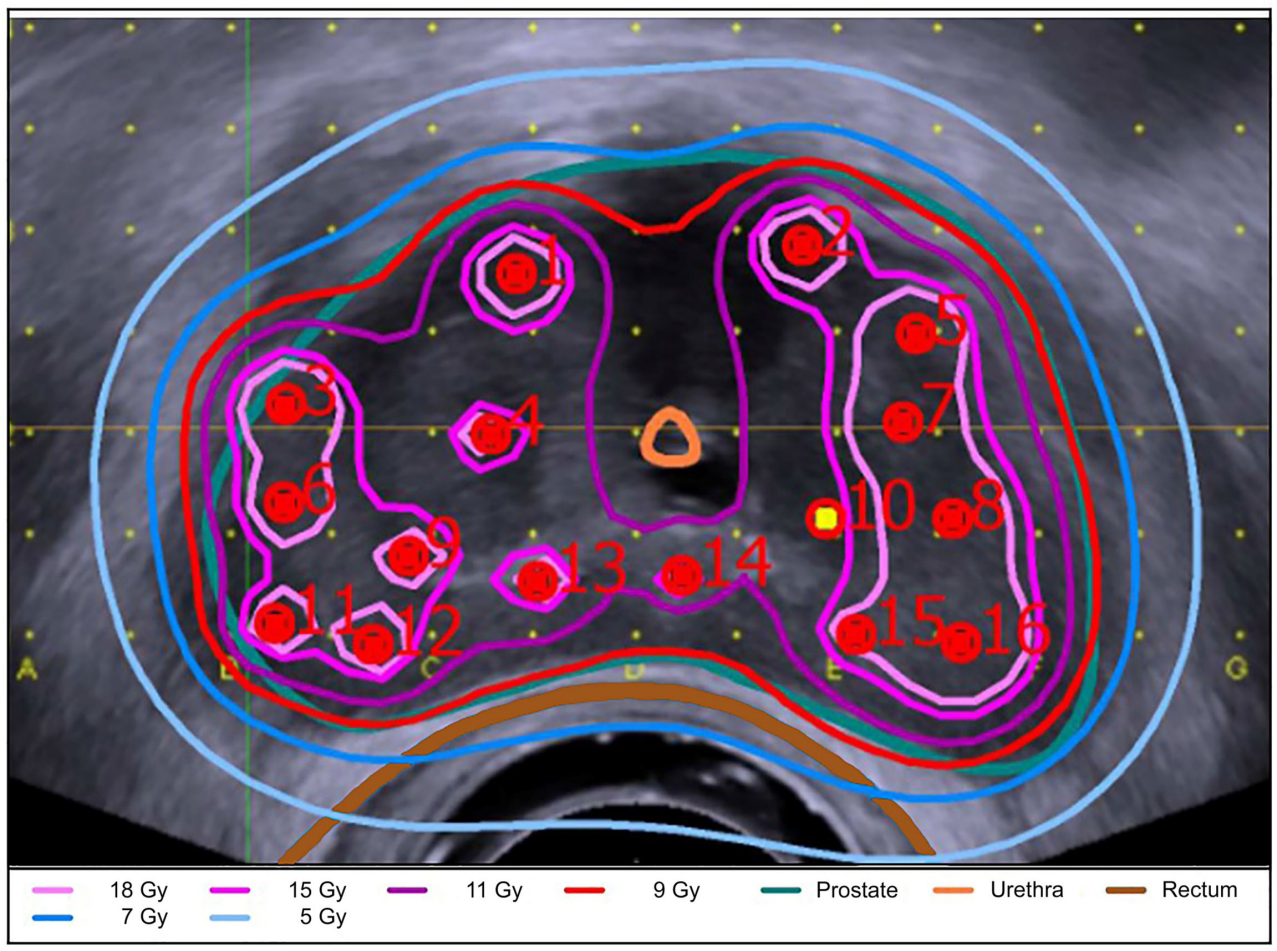
3D TRUS-supported intraoperative radiation planning. Shown is the 3D TRUS-supported intraoperative radiation planning using the SagiPlan treatment planning system (Eckert & Ziegler BEBIG GmbH). The prostate (turquoise), rectum (brown), intraprostatic urethra (orange) and the isodose distribution are shown in an axial view. The isodose distribution is coded with the following colours: light pink = 18 Gy (D_180%_); pink = 15 Gy (D_150%_); purple = 11.0 Gy (D_110%_); red = 9 Gy (D_ref_); blue = 7 Gy (D_70%_); light blue= 5 Gy (D_50%_).

Approximately two weeks after completion of EBRT, two HDR-BT boost fractions were performed with a 14-day interval between the two applications. Each session required new implantation. **Figure 1** illustrates the timing and sequence of brachytherapy. Transperineal brachytherapy catheter implantation was performed with 3D TRUS-guided online planning in lithotomy position in general or spinal anesthesia by a small, limited group (n = 3) of brachytherapy experts. In 2008, the brachytherapy source was changed from Ir-192 to Co-60. As equipment for HDR-BT applications, the Multi-Source and SagiNova HDR afterloader (Eckert & Ziegler BEBIG GmbH) in combination with the treatment planning systems HDRplus, SagiPlan (Eckert & Ziegler BEBIG GmbH), and Nucletron PLATO were used. The HDR-BT boost PTV was defined as the entire prostate without the seminal vesicles and additional margin. The prescription dose for the PTV was 9 Gy (D_90%_) per fraction. The proportion of the PTV receiving 150% (V_150%_) should be below 50% and the V_200%_ below 25%. The maximum dose to the urethra was kept below 13 Gy (D_Max_) and to the rectum below 9 Gy. An example of the 3D TRUS-supported intraoperative radiation planning is shown in **Figure 2**. The combined EBRT and HDR-BT boost resulted in a biologically effective dose (BED) of 233.33 Gy and an equivalent dose in 2 Gy fractions (EQD_2_) of 100 Gy using an α/β-value of 1.5 Gy.

### Statistics

Biochemical relapse-free survival, overall survival, prostate-specific survival, and freedom from distant metastasis were determined by the Kaplan–Meier method with associated log-rank testing for significant differences. Cox regression hazard model was applied for univariate and multivariate analyses adjusted to initial PSA, TNM stage, androgen deprivation therapy, and Gleason score. Differences were considered statistically significant in the case of a two-sided p-value of < 0.05. Statistical analysis was conducted using IBM SPSS v.26.0 (IBM Corp., Armonk, NY, USA).

## Results

The median follow-up of the whole cohort, consisting of 338 patients, was 101.8 (range 0.2–230.7) months. Classified by D’Amico, 15, 115, and 208 patients had low-, intermediate- and high-risk prostate cancer, respectively (21). Total treatment time was median 62 days (range 45-125 days) with a median time to first HDR-BT fraction of 14 days (range 2-76 days) after EBRT. Clinical characteristics are summarized in **Table 1**.

**Table 1:** Patient and treatment characteristics.

Characteristics	(n=338)
**Median age in years (range)**	69.0 (50.0-81.3)
**Median KPS in % (range)**	90 (30-100)
**Median iPSA in ng/mL (range)**	10.1 (0.4-233.0)
**iPSA group**	
**< 10 ng/mL**	164 (48.5%)
**10-20 ng/mL**	88 (26.0%)
**> 20 ng/mL**	86 (25.4%)
**N/A**	0 (0%)
**Gleason-Score**	
**≤ 6**	63 (18.6%)
**7a**	102 (30.2%)
**7b**	71 (21.0%)
**8 - 10**	100 (29.6%)
**N/A**	2 (0.6%)
**T-Stage**	
**T1**	154 (45.6%)
**T2**	116 (34.3%)
**T3**	68 (20.1%)
**T4**	0 (0%)
**N/A**	0 (0%)
**D`Amico risk group**	
**Low-risk**	15 (4.4%)
Intermediate-risk	115 (34.0%)
**High-risk**	208 (61.5%)
N/A	0 (0%)
**Lymph node irradiation**	116/338 (34.3%)
**Low-risk**	4/15 (26.7%)
Intermediate-risk	17/115 (14.8%)
**High-risk**	95/208 (45.7%)
**Androgen deprivation therapy**	176/338 (52.1%)
**Low-risk**	6/15 (40%)
Intermediate-risk	36/115 (31.3%)
**High-risk**	134/208 (64.4%)
**Imaging before treatment**	
**MRI**	79 (23.4%)
**PET-CT**	19 (5.6%)
**Treatment technique**	
3D conformal	205 (60.7%)
**IMRT**	27 (8.0%)
**VMAT**	106 (31.4%)

KPS, Karnofsky Performance Status; N/A, not available; iPSA, initial prostate-specific antigen; 3D-CRT, 3D-conformal radiation therapy; IMRT, intensity-modulated radiation therapy; VMAT, volumetric-modulated arc therapy.

**Figure 3:**
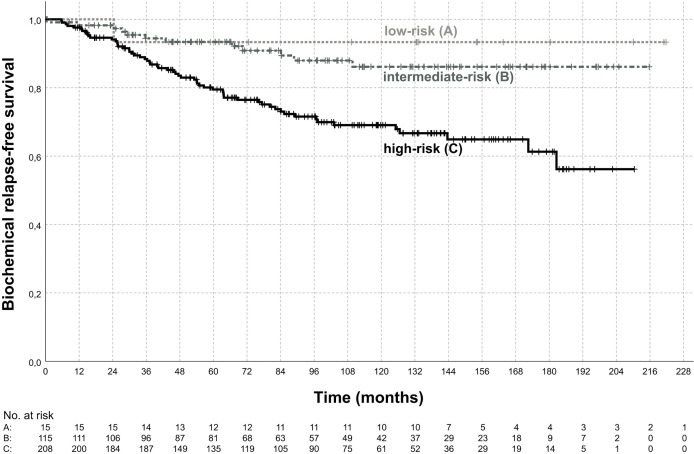
Biochemical relapse-free survival. Shown is the biochemical relapse-free survival according to risk group for the low-risk group **(A)**, intermediate-risk group **(B)**, and high-risk group **(C)**. The estimated biochemical relapse-free survival at 5-years was 93.3%, 93.4%, 79.5% for low-, intermediate-, high-risk disease, respectively. Biochemical relapse-free survival was significantly different between intermediate-risk and high-risk (p < 0.01, log-rank test).

72 (21.3%) patients developed a biochemical relapse and in 37 (10.9%) patients distant metastases occurred during follow-up. The estimated biochemical relapse-free survival (bRFS), freedom from distant metastasis (FFM), and overall survival (OS) at 5 years were 84.7%, 93.4%, and 90.1%, respectively. At 10 years the estimated bRFS, FFM, and OS in our patient sample were 75.9%, 86.5%, and 70.0%, respectively. **Figure 3** shows the bRFS for each risk group according to the D`Amico classification.

Parameters for Cox regression analyses were TNM stage (≤T2b; ≥T2c), Gleason score ≤7a (3 + 4) *versus* ≥7b (4 + 3), initial PSA (continuous variable), ADT, Age (continuous variable), and MRI before treatment. Gleason score was found to be a prognostic factor for bRFS, FFM, and OS in both univariate and multivariate analyses. Initial PSA was a significant prognostic factor in multivariate analysis for bRFS, but not for FFM and OS. In multivariate analysis, TNM stage was prognostic for FFM, but not for bRFS and OS. ADT was not prognostic for bRFS, FFM, and OS in multivariate analysis in the whole patient cohort, in the intermediate-risk group, and the high-risk group. MRI was not prognostic for any outcome parameter. Age was a significant prognostic factor for OS. PSA kinetics were not available for analysis and, therefore, we cannot exclude them from being residual confounders. The results of the Cox regression analysis are summarized in **Table 2**.

**Table 2:** Cox regression analysis.

Variable		BRFS (N = 332; N/A = 6)			FFM (N = 331; N/A = 7)			OS (N = 334; N/A = 4)		
	N	HR	95% CI	P	HR	95% CI	P	HR	95% CI	P
**TNM**										
<T2C	222	1.00	0.96-2.53	0.07	2.07	1.03-4.17	0.04	1.00	0.72-1.52	0.82
≥T2C	116	1.56						1.05		
**GLS**										
<7b	165	1.00	1.77-5.18	< 0.01	5.29	2.24-12.52	< 0.01	1.00	1.19-2.57	< 0.01
≥7b	171	3.03						1.75		
**iPSA (cv)**	338	1.01	1.00-1.01	0.01	1.00	0.99-1.01	0.51	1.00	1.00-1.01	0.64
**ADT**										
No	162	1.00	0.50-1.41	0.51	0.95	0.46-1.99	0.90	1.00	0.76-1.63	0.60
Yes	176	0.84						1.11		
**MRI**										
No	259	1.00	0.47-1.66	0.70	0.57	0.20-1.61	0.29	1.00	0.35-1.41	0.32
Yes	79	0.88						0.70		
**Age (cv)**	338	0.99	0.96-1.03	0.73	0.97	0.93-1.02	0.30	1.04	1.01-1.07	0.02

bRFS, biochemical relapse-free survival; FFM, freedom from distant metastasis; OS, overall survival; iPSA, initial prostate-specific antigen; N/A, not available; HR, hazard ratio; CI, convidence interval; CV, continuous variable.

**Figure 4:**
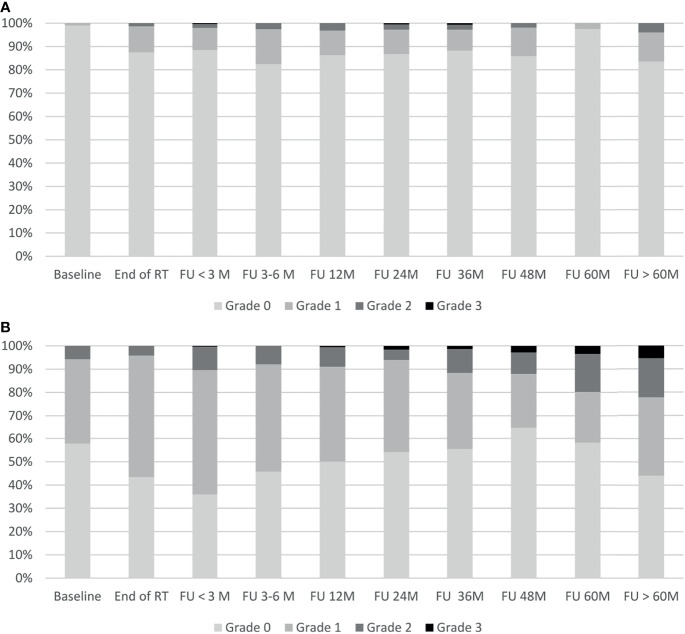
Gastrointestinal and genitourinary toxicity. Shown is the time course of physician-recorded gastrointestinal toxicity **(A)** and genitourinary toxicity **(B)** according to CTCAE v5.0. RT, radiotherapy; M, months; FU, follow-up.

The temporal occurrence of GI and GU toxicity is shown in **Figure 4**. Late grade 2 GI toxicity peaked at the 12-month follow-up, decreased thereafter, and showed a second peak in the very late follow-up period after 60 months. No grade 4 GI toxicities were observed. One patient with rectal hemorrhage developed late grade 3 (0.3%) GI toxicity cumulated over 5 years of follow-up. Overall, a cumulative 5-year late GI toxicity grade ≥ 2 was observed in 5.0% of the patients. Late grade 2 to 3 GU toxicity showed a constant increase from the 24-month follow-up until the very late follow-up period after 60 months of follow-up. No grade 4 toxicities were observed. After 5 years of follow-up, 12 patients (3.6%) developed late grade 3 GU toxicity: All 12 patients suffered from late grade 3 urinary tract obstruction with 1 out of 12 developing additional grade 3 non-infective cystitis and urinary incontinence. Overall, a cumulative 5-year late GU toxicity grade ≥ 2 was observed in 19.3% of the patients.

## Discussion

Dose-escalation has demonstrated the ability to increase biochemical control in the management of prostate cancer. In this context, HDR-BT boost offers the possibility of highly conformal dose-escalation with excellent adjacent organ at risk sparing and has compared favorably to EBRT alone in the literature (6, 22–24). Our analysis differs from other published data by the applied treatment schedule: HDR brachytherapy was applied sequentially two and four weeks after EBRT, resulting in a total treatment time of median 62 days (**Figure 1**). A strength of the presented study is the absence of changes in the target volume definition or fractionation scheme, as all patients were treated with a standardized protocol. The matured median follow-up of 101.8 months, therefore, allows a comparison to updated randomized long-term data on HDR-BT boost: Hoskin et al. investigated hypofractionated EBRT alone or in combination with HDR-BT boost: treatment was randomized to 55 Gy in 20 fractions or 35.75 Gy in 13 fractions with 17 Gy HDRBRT boost in two fractions. A statistically significant difference in biochemical failure-free survival was demonstrated in favor of the combined modality and remained significant in the 12-year data update. GU and GI toxicity was not significantly different between both treatment arms (6). Sathya et al. randomized combined 35 Gy low-dose-rate (LDR) brachytherapy with EBRT of 40 Gy in 20 fractions *versus* EBRT of 66 Gy in 33 fractions. Biochemical control was improved for the combined treatment arm but failed to reach statistical significance in a recent update (22, 23). Looking beyond HDR-BT boost, the ASCENDE-RT trial compared dose-escalated EBRT of 78 Gy to EBRT of 46 Gy combined with 115 Gy LDR brachytherapy boost (25). 7-year biochemical failure-free survival in the LDRBT boost arm was 86% and 75% in the EBRT arm and therefore significantly increased for the combination therapy. Late GU toxicity was increased with 5-year grade 3 GU toxicity of 18.4% for LDRBT boost and 5.2% for the EBRT-only arm (p < 0.001). Recently, the phase 2 RTOG 0321 trial reported the results of 45 Gy EBRT in 25 fractions in combination with 19 Gy HDR-BT boost in two fractions within 24 hours: Biochemical failure rates per Phoenix definition at 5 and 10 years were 14% and 23% and the cumulative grade 3-5 GU/GI toxicity was 4% at 5 years (26).

Our outcome data is comparable to large retrospective analyses and randomized trials with a reported estimated 5-year bRFS, FFM, and OS of 84.7%, 93.4%, 90.1%, respectively for all patients and estimated 5-year bRFS of 79.5% for high-risk patients. Cumulative 5-year late grade 3 GU/GI toxicity occurred in 3.6/0.3% of the patients and is within the range of reported late toxicity incidence of randomized HDR-BT boost trials (6, 23, 26). In the present study, no evidence of compromised biochemical control by two-weekly HDR-BT boost after EBRT and the resulting long treatment time could be detected compared to the literature (**Supplementary Table 1**). The assumed proliferation equivalent of 0.24 Gy per day for EBRT alone might play a subordinate role when ultra-high single doses are used, as in HDR brachytherapy or stereotactic body radiotherapy (27, 28).

Currently, there is a general trend to shorter treatment courses by reducing the number of HDR brachytherapy fractions. We chose to implement two fraction HDR-BT boost in two separate sessions to improve patient compliance and to reduce the risk of catheter displacement compared to two brachytherapy fractions within 24 hours. Furthermore, in the monotherapy setting, single fraction HDR brachytherapy was recently shown to be inferior to two fraction HDR brachytherapy by Morton et al. (29).

The role of additional ADT in prostate brachytherapy remains debatable as the literature shows heterogeneity. A recent network meta-analysis of randomized trials by Jackson et al. showed an 88% probability that EBRT combined with ADT leads to an improved OS compared to EBRT combined with brachytherapy in intermediate- and high-risk disease (30). On the other hand, a systematic literature overview of the American Brachytherapy Society Task Group, including 52 studies with 43303 patients, showed no benefit for the addition of ADT to brachytherapy in low-risk and favorable intermediate-risk patients as well as most HDR brachytherapy trials (31). Keyes et al. observed an improvement in biochemical progression-free survival of up to 15% for the addition of ADT to brachytherapy for unfavorable intermediate- and high-risk patients as well as patients with suboptimal dosimetry at the cost of a potential overall survival detriment (31). Consistent with a large number of retrospective studies, our data did not demonstrate a benefit of additional ADT in bRFS, FFM, and OS in multivariate Cox regression analysis for the whole patient cohort, the intermediate-risk group as well as the high-risk group.

The findings from our retrospective study require further investigation in randomized controlled trials. Nevertheless, our analysis demonstrated promising biochemical control and low toxicity rates for two-weekly HDR-BT boost after EBRT.

## Conclusions

Two-weekly HDR brachytherapy boost after EBRT for localized prostate cancer is safe and feasible. With excellent biochemical control and low rates of gastrointestinal and genitourinary toxicities, two-weekly HDR brachytherapy boost can be considered as a standard treatment regime in clinical practice. The addition of ADT to combined HDR-BT boost and EBRT did not improve clinical outcome.

## Data Availability Statement

The raw data supporting the conclusions of this article will be made available by the authors, without undue reservation.

## Ethics Statement

Ethical review and written informed consent was not required for participation in this retrospective analysis in accordance with the local legislation [BayKrG Art. 27 (4)] and institutional requirements. Written informed consent for treatment and retrospective data analysis was provided by all patients before start of treatment.

## Author Contributions

JT, MF, and BP contributed to conception and design of the study. JT, PL, DL, and SW organized the database. JT performed the statistical analysis. JT wrote the first draft of the manuscript. PL and JK performed critical revision of the article for important intellectual content. All authors contributed to manuscript revision, read, and approved the submitted version.

## Funding

This publication was supported by the Open Access Publication Fund of the University of Wuerzburg.

## Conflict of Interest

The authors declare that the research was conducted in the absence of any commercial or financial relationships that could be construed as a potential conflict of interest.

## Publisher’s Note

All claims expressed in this article are solely those of the authors and do not necessarily represent those of their affiliated organizations, or those of the publisher, the editors and the reviewers. Any product that may be evaluated in this article, or claim that may be made by its manufacturer, is not guaranteed or endorsed by the publisher.
