# Supplementary material for: Two-Weekly High-Dose-Rate Brachytherapy Boost After External Beam Radiotherapy for Localized Prostate Cancer: Long-Term Outcome and Toxicity Analysis
Source: Front Oncol. 2021 Nov 26;11:764536. doi: 10.3389/fonc.2021.764536 (PMC8660669; doi:10.3389/fonc.2021.764536)
Supplement: Supplementary file 1 [file DataSheet_1.docx]

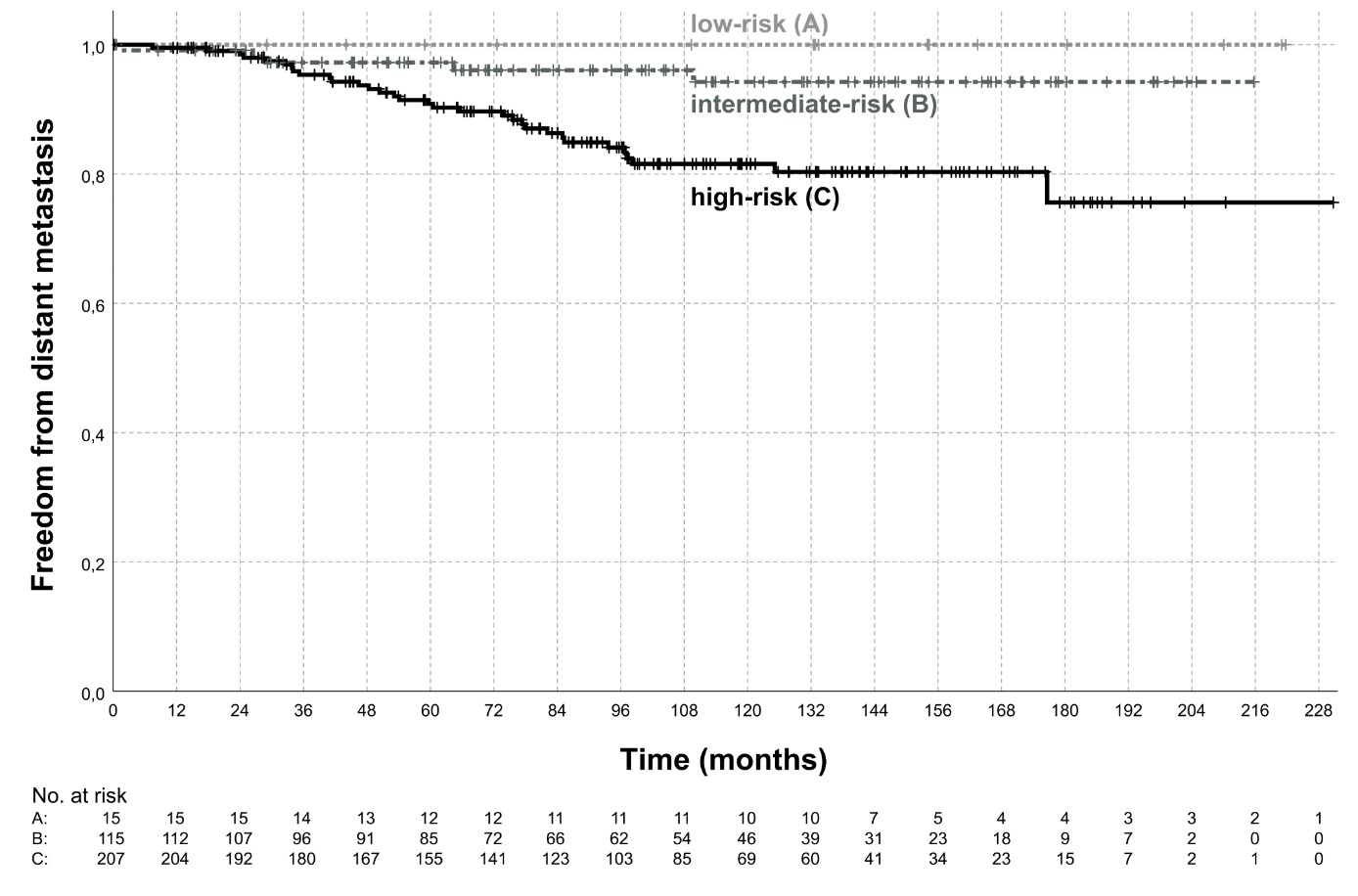


**Supplementary Fig. 1: Freedom from distant metastasis.**

Shown is the freedom from distant metastasis for the low-risk group (A), intermediate-risk group (B) and the high-risk group (C). The estimated freedom from distant metastasis at 5-years was 100.0%, 97.2%, 90.9% for low-, intermediate-, high-risk disease, respectively. Freedom from distant metastasis was significantly different between intermediate-risk and high-risk (p < 0.01, log-rank test).
