# Supplementary material for: Two-Weekly High-Dose-Rate Brachytherapy Boost After External Beam Radiotherapy for Localized Prostate Cancer: Long-Term Outcome and Toxicity Analysis
Source: Front Oncol. 2021 Nov 26;11:764536. doi: 10.3389/fonc.2021.764536 (PMC8660669; doi:10.3389/fonc.2021.764536)
Supplement: Supplementary file 2 [file Table_1.docx]

**Supplementary Table 1: Literature overview**

| **First author** | **n** | **HDR** | | **EBRT** | | **FU (y)** | **Biochemical recurrence-free survival (%)** | | | | | **Grade 3-4 toxicity (%)** | |
| --- | --- | --- | --- | --- | --- | --- | --- | --- | --- | --- | --- | --- | --- |
|  |  | **total dose (Gy)** | **Fx** | **total dose (Gy)** | **Fx** |  | **All** | **LR** | **IR** | **HR** | **y** | **GU** | **GI** |
| Martell 2019 | 518 | 15 | 1 | 37.5 | 15 | 5.2 |  |  | 91 |  | 5 | 5 | 0 |
| Hoskin 2012/2021 | 218 | 17 | 2 | 35.75 | 13 | 10.9 | 71 |  |  |  | 5 | 26^†^ | 7^†^ |
| Kasahara 2020 | 66 | 18 | 2 | 39 | 13 | 4.4 |  |  |  | 89 | 5 | 3 | 0 |
| Martinez 2011 | 472 | 16.5-23 | 2-3 | 46 | 23 | 8.2 | 81.1 |  |  |  | 10 | 2-3 | <0.5 |
| Prada 2012 | 252 | 23 | 2 | 46 | 23 | 6.2 |  |  |  | 84 | 5 | 1.2 | 0 |
| Galalae 2014 | 122 | 18* | 2 | 40 | 25 | 9.7 | 77.8 |  |  |  | 5 | 4.9 | 2.5 |
| Deger 2005 | 442 | 18-20 | 2 | 40-50.4 | 20-28 | 5 | 65** | 81** | 65** | 59** | 5 | 11 | 1 |
| Neviani 2011 | 455 | 16.5-21 | 3 | 45 | 25 | 4.0 |  | 92 | 88 | 85 | 5 | 8 | 0.6 |
| Savdie 2012 | 90 | >16.5 | 3 | 45 | 25 | 7.9 |  |  |  | 80 | 5 |  |  |
| Phan 2007 | 309 | 22-24 | 4 | 45 | 25 | 4.7 |  | 98 | 90 | 78 | 6 | 4 | 0.3 |
| Demanes 2005/2009 | 411 | 22-24 | 4 | 36-39.6 | 20-22 | 6.4 |  | 92 | 87 | 63 | 10 | 7.7 | 0 |
| Strouthos 2018 | 303 | 21 | 2 | 45 | 25 | 6.0 | 88.3 |  |  | 85.6 | 7 | 2.5 | 0 |
| Vigneault 2017 | 832 | 15-21 | 1-3 | 36-45 | 12-25 | 5.5 | 94.6 | 94.8 | 95 | 93.5 | 5 | 6.6 | 0 |
| Present study | 338 | 18 | 2 | 46 | 23 | 8.4 |  | 93.3 | 93.4 | 79.5 | 5 | 3.6 | 0.3 |

*prescribed as 30 Gy in 2 fractions to the peripheral zone; **progression-free survival; †classification after Dische et al. (1989)

Abbreviations: n= number of patients; y = years; Fx = fractions; FU = follow-up; LR = low-risk; IR = intermediate-risk; HR = high-risk; GU = genitourinary toxicity; GI = gastrointestinal toxicity.
